# Presence of white-nose syndrome in bats from Southern Mexico

**DOI:** 10.1371/journal.pone.0318461

**Published:** 2025-05-19

**Authors:** Gabriela Elena Medina-Cruz, Carla Gabriela May-Mutul, Paola Nicté López-González, Lizbeth Josefina González-Herrera, Javier Enrique Sosa-Escalante, Angel Rodríguez-Moreno, Judith Castellanos-Moguel, Héctor David Martínez-Tamayo, Gabriel Gutiérrez-Granados, Víctor Sánchez-Cordero, Miguel Briones-Salas

**Affiliations:** 1 Laboratorio de Vertebrados Terrestres (Mastozoología), Centro Interdisciplinario de Investigación para el Desarrollo Integral Regional (CIIDIR-Oaxaca), Instituto Politécnico Nacional, Calle Hornos, Col. Nochebuena, Santa Cruz Xoxocotlán, Oaxaca, México; 2 Grupo DIMYGEN-CEGES, Diagnósticos Moleculares y Genéticos (DIMYGEN Laboratorio) y Centro para la Gestión de la Sustentabilidad (CEGES), Calle 78 num. 578 entre 13-1 y 128 Residencial Pensiones, Mérida, Yucatán, México; 3 Laboratorio de Genética, Centro de Investigaciones Regionales “Dr. Hideyo Noguchi”, Universidad Autónoma de Yucatán. Mérida, Yucatán, México; 4 Pabellón Nacional de Biodiversidad, Departamento de Zoología, Instituto de Biología, Universidad Nacional Autónoma de México, CDMX, México; 5 Laboratorio de Micología, Departamento El Hombre y Su Ambiente, Universidad Autónoma Metropolitana-Xochimilco, Calzada del Hueso, Villa Quietud, CDMX, Mexico; 6 UMIEZ, Facultad de Estudios Superiores Zaragoza, UNAM, Batalla 5 de mayo s/n esquina Fuerte de Loreto, Col. Ejército de Oriente, Iztapalapa, CDMX, México; National Veterinary Research Institute (NVRI), NIGERIA

## Abstract

White-nose syndrome (WNS), caused by the fungus *Pseudogymnoascus destructans,* is increasingly causing high mortality in North American vespertilionid bats. This fungus has become widely established, appearing in bat populations from Asia, Europe and North America, including in the state of Texas, U.S., creating a high potential for dispersal into neighboring Mexico. In this study, we collected samples from 11 captured individuals and 15 carcasses of *Myotis velifer, Dermanura azteca, Pteronotus parnellii, Desmodus rotundus, Balantiopteryx plicata* and *Anoura geoffroyi* species of bat that were living in a cave in southern Mexico. Using morphological and molecular techniques, we found *P. destructans* in vespertilionid and emballonurid bats, including 9 individuals of *M. velifer* (8) and *P. parnellii* (1), and in 1 carcass of *B. plicata*. Captured individuals and carcasses showed injuries mostly to their wings, patagium and rostrum. Thermotolerance experiments confirmed that *P. destructans* can grow at a wide range of temperatures (5–28 °C), making this fungus a risk to bat species in a wide range of habitats, including the tropical environments of southern Mexico. This study evidences the presence of *P*. *destructans* in southern Mexico, validating the need for a monitoring program and education to inform communities of the potential detrimental that *P. destructans* may have on other populations and species of bats in a Mesoamerican biodiversity hotspot.

## Introduction

The proliferation of emerging diseases affecting wildlife has raised concerns worldwide [1,2]. Emerging diseases have become particularly troublesome when hosts and vectors disperse pathogens to new habitats, expanding risk to other areas, and exposing other potential species to infections [1,3,4]. Several studies have documented that dispersal of pathogens by hosts, vectors and/or human activities ranges from local to distant geographical areas, spreading across continents [1–5]. The geographical scale of pathogen dispersal is closely related to the vagility of their hosts and vectors. For example, some pathogens are restricted to host (e.g., rats and mice) and vector (e.g., some insects) species with a limited dispersal capability, which makes a rapid long-range dispersal to new areas difficult [1,2]; however, pathogens that are carried by host and/or vector species with high dispersal capabilities, such as migratory species, have a much wider dispersal capacity, infecting susceptible populations along the way. When these areas have high biodiversity, losses are amplified in both present and future populations because zoonotic diseases are known to lay dormant and then re-emerge [6–10].

The rapidly spreading disease white-nose syndrome (WNS) in Asia, Europe and North America, caused by the fungus *Pseudogymnoascus destructans* [5], is becoming a significant threat to vespertilionid bat populations in North America. An estimated 5 million bats have died from WNS in North America alone [11–14]. This fungal pathogen invades deep into skin tissue, affecting the wings, patagium and rostrum of various species of bats and is transmitted through direct contact between individuals, a contaminated environment and/or ectoparasites during the fall [15]. These infections progress through the winter as bats hibernate in underground sites (such as caves, mines, and tunnels) from late fall to early spring. This fungal pathogen that grows on bats’ exterior is often not visible, but the infection causes bats to awaken from hibernation during the winter when food supplies are low, prompting the use of their meager fat reserves, resulting in a disruption to their water and electrolyte balance and causing death by starvation and dehydration [11–14,16].

Humans traveling from Europe to North America are likely dispersers of the fungus that causes WNS [17]. *P. destructans* was first documented in North American in Albany, New York, U.S. in 2006 [17] and has since spread rapidly throughout the U.S. and Canada [17–20]. In 2018, the closest recorded outbreak of WNS was reported in San Antonio, Texas, U.S. [21,22]. The capacity of this fungus to thrive in climates across a wide range of temperatures (5–28 °C) [19,23,24] and its ease of dispersal via migratory species of bats and other hosts makes WNS a major threat to bat populations (see below).

*P. destructans* is not species-specific, having been documented to infect and cause harm to species of vespertilionid bats as *Myotis grisescens, Myotis sodalis, Myotis lucifugus, Myotis septentrionalis, Myotis leibii, Myotis velifer, Myotis thysanodes, Myotis volans, Perimyotis subflavus* and *Eptesicus fuscus* [25,26], and also be hosted by species of bats as *Lasiurus borealis, Tadarida brasiliensis, Corynorhinus rafinesquii, Lasionycteris noctivagans, Corynorhinus townsendii, Corynorhinus townsendii virginianus, Corynorhinus townsendii ingens* and *Myotis ciliolabrum* although without evidently causing harm to the individuals who occupy a disperser role [26]. A previous study used ecological niche modeling to project the potential distributions of bats and *P. destructans* fungi strains into Mexico [27,28]. Ten species of bats and *P. destructans* fungi strains overlapped their potential distribution in Mexico, including high-elevation areas in the Sierras of the northwest (Sierra Madre Occidental) and northeast (Sierra Madre Oriental), the Transvolcanic Belt of central Mexico and southwest (Sierra Madre del Sur) regions nationwide. The 10 species of bats included *C. townsendii, E. fuscus, Lasiurus cinereus, L. noctivagans, M. ciliolabrum, Myotis evotis, M. lucifugus, M. velifer, M. volans* and *Myotis yumanensis*. Moreover, the recent modeling of the ecological niche of *P. destructans* maps its entry route into Mexico, further evidencing the potential presence of this harmful fungus in the country. We can expect *P. destructans* to thrive in the highlands of southern Mexico because the climate resembles environments where WNS has been recorded in some regions of North America [27,28].

The State of Oaxaca is a Mesoamerican biodiversity hotspot [29] that holds the highest species richness of bats in Mexico with 96 species [30]. Recently, it has been reported that bats show lesions in their wings and body, apparently caused by fungal pathogens, such as *P. destructans* in Oaxaca [31]. During field work in the Sierra Mixteca region in Oaxaca, we collected individuals and carcasses of *Pteronotus parnellii, Anoura geoffroyi, Desmodus rotundus, Balantiopteryx plicata, M. velifer* and *Dermanura azteca* bat species collected from inside a cave to test for the presence of *P. destructans* in the population and to measure the thermotolerance of the fungus when grown under laboratory conditions and compare that with the temperature range measured in our study site.

## Materials and methods

### Study area

**Fig 1:**
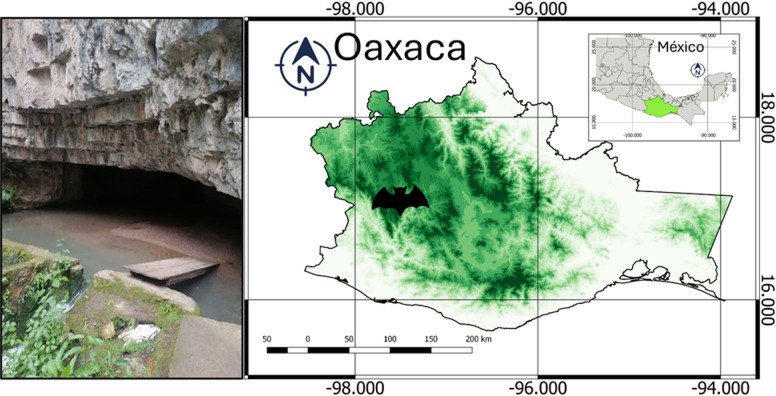
Map showing the location of the study site in Oaxaca (bat on map), Southern Mexico. A view of the entrance of the study site cave where individuals and carcasses of species of bats were collected.

A cave called Kava Yuu Yavi (“grief with a hole of water” in Mixtec language) located in the municipality San Pedro de Los Molinos, Oaxaca, Southern Mexico, served as the study site (Fig 1). The climate in this area is predominantly temperate subhumid, with the rainy season extending from May to October and the dry season spanning November to April [32]. Several types of vegetation have been identified in the region, including juniper forests, deciduous forests, oak woodlands, pine forests, chaparral, thorny shrubland, and lowland deciduous forest [33]. The temperature ranges from 14–20 °C, and the elevation ranges from 1900–2800 m a.s.l. [34]. The average microclimatic values recorded (February to June) at the entrance and at the back inside the cave were as follows: wind, 0 m/s; average temperature, 19.85 °C; humidity, 69.3%, heat index, 19.25 °C; dew point, 13.6 °C, and evaporation point 15.7 °C.

### Sampling protocol

Samples of captured individuals and carcasses of bats were collected in February and March 2018, February and April 2020 and February 2023. We followed handling protocols for wild mammals, based on recommendations from the American Association of Veterinary Surgeons [35] and according to the guidelines of the American Society for the Use of Mammalogists of Wild Mammals in Research [36], and under a collecting permit issued by the General Direction of Wildlife of Mexico (permission numbers SGPA/DGVS/04283/17, SGPA/DGVS/010680/18, SGPA/DGVS/02142/bitácora 09/22). Individuals were captured using mist nests (12 m long x 2.6 m high) set at the entrance of the cave for eight hours and checked every 30 m. Inside the cave, we used type 5 suits, latex gloves, N95 masks (2–5 μm) and glasses for protection against exposure to aerosols and spores. Individual bats with signs of the presence of fungi on their wings and bodies were captured. Each bat identified with lesions was assigned a number from 0–5 to indicate the severity of wing lesions according to the scale proposed [11]. Bats were assigned 0 to indicate no apparent macroscopic lesions; 1 for the presence of spots, patches of depigmented membranes; 2 for flaking and depigmentation of the forearm; 3 for tissue necrosis; 4 for wing perforation; and 5 for membrane loss. Only individuals with a wing damage index of 2 or higher were included in the study. Once the samples were obtained, the individuals were released at the same site where they were captured. Carcasses were collected from the floor of the cave in paper bags to avoid humidity and bacterial contamination. The species and sex of bats were annotated. We acknowledge that eliminating contamination from fungi spores in live bats is challenging, as it would require direct intervention in the cave’s environment or in individual bats, which could be highly disruptive.

Fungi were collected by smashing sterile swabs humidified with sodium chlorine directly from fungi colonies living on captured individuals and carcasses (S1 File, Supplemental Information). Swabs were individually stored inside a conic sterile microtube of 1.5 ml and marked for identification. We extracted fungi samples from the patagium (plagio-patagium, dactyl-patagium, and uropatagium). The first sample was stored in formol at 10% for histopathological analysis [37] and a second sample was stored at −20 °C for molecular analyses. A total of 11 samples from captured bats and 15 swab samples from bat carcasses were taken to the DIMYGEN (Genetic and molecular diagnosis laboratory) of the DIMYGEN-CEGES research group for processing. Fieldwork was performed following the decontamination protocol for WNS issued by http://whitenosesyndrome.org/ North America’s Response to the Devastating Bat Disease. To detect the fluorescence of bat wings in response to long-wave ultraviolet light (S1 File), we used a small hand-held E T EASYTAO brand lamp, UV flashlight, 395 nm ultraviolet UV LED lamp [38].

### Molecular analyses and histopathological analyses

It was decided to euthanize several infected specimens using isoflurane overdose as recommended by American Association of Veterinary Surgeons [35]. Two tissue samples of approximately 1 cm² were dissected from sacrificed individuals and carcasses where fungi colonies were visually abundant. One tissue sample was placed in 10% formaldehyde; the wing membrane was stained with haematoxylin and eosin for histopathological analyses [37]. A second tissue sample was stored in 1.5 ml conic microtubes and marked.

DNA was extracted from dissected tissue and swab samples using the commercial kit Quick-DNA™ Fecal/Soil Microbe Microprep (Zymo Research). Samples were processed one by one to avoid cross contamination. The extracted DNA was quantified by fluorescence using the kit Fluorometer Quantus® (Promega) of the system QuantiFluor® ONE dsDNA (Promega). To validate the presence of *P. destructans*, we amplified fragments of 624 bp of the internal transcript region (ITS) of the RNA ribosomal gene with the following oligonucleotides: nu-SSI (1506)-184–5’-Gd Fw (5’-GGG GAC GTC CTA AAG CCT-3’) and nu-5.8S-144-3’-Gd Rv (5’-TTG TAA TGA CGC TCG GAC-3’) were amplified [39]. The reactions had concentrations in a final volume of 17 µl: 7.5 µl GoTaq Green Master Mix (Promega), 0.4 µl of each oligonucleotide, 5.7 µl nucleases free water and 3 µl of DNA. The conditions of the thermal cycler Arktik Thermal Cycler (Thermo Scientific) were standardized using negative controls and positive controls of *P. destructans* (strain ATCC MYA 4855, purchased from the American Type Culture Collection) and were as follows: one cycle of 10 min at 98 °C, followed by 40 cycles at 95 °C for 30 seconds, at 52 °C for 35 seconds, at 72 °C for 40 seconds, respectively, and one cycle at 72 °C for 10 minutes. Under these conditions, a 624 bp fragment corresponding to *P. destructans* positive control was successfully amplified. Collected samples were amplified one by one using negative and positive controls in each PCR. Positive PCRs were amplified a second time to avoid false-positive results.

Amplified fragments were purified from agarose gel cuts using the commercial kit Zymoclean™ Gel DNA Recovery (Zymo Research). The purified amplicons were quantified with the Fluorometer Quantus® of Promega and conducted on the system QuantiFluor® One dsDNA. The cyclo sequencing of the positive fragments were performed using a commercial kit BigDye® Terminator v3.1 Cycle Sequencing (Applied Biosystem) and the thermal cyclo Arktik Thermal Cycler (Thermo Scientific) under the following conditions: 25 cycles for each treatment at 96 °C for 1 minute, at 96 °C for 10 seconds, at 50 °C for 5 seconds, and at 60 °C for 4 minutes, respectively. Samples were purified following cyclo sequencing using the commercial kit BigDye® X-Terminator™ (Applied Biosystem). The fragments of each sample were sequenced in direction F and counter-direction R by capillary electrophoresis using a genetic analyzer SeqStudio (Applied Biosystem). The sequences obtained were analyzed with the software Sequencing Analysis Version 6 (Applied Biosystem) and the program MEGA version 11® (https://www.megasoftware.net/). These were aligned with other sequences in GenBank® using the algorithm Megablast (https://www.ncbi.nlm.nih.gov/) for identifying similar sequences.

### Thermotolerance

Due to the higher temperature ranges of the location where the fungus was collected, thermotolerance tests were performed with a *P. destructans* reference strain of ATCC. *P. destructans* ATCC MYA 4855 strain was activated on Sabouraud Dextrose Agar, and the macro- and micromorphology were verified (S2 File). Due to the slow growth of the *P. destructans* ATCC MYA 4855 strain, it was cultured at 5 °C (temperature for psychrophiles), 17 °C, 20 °C and 28 °C in Sabouraud Dextrose Agar (SDA) for 60 days (Bioxón Mexico). These temperature ranges have been reported to be close to optimum values in other pathogenic fungi [40]. A 4 mm mycelium disk was placed in the center of a Petri dish; the minimum and maximum diameter were measured daily, and the micromorphology was verified daily and at the end of the assays. The experiments were performed in triplicate on different dates, and the average of the measurements was reported.

## Results

**Fig 2:**
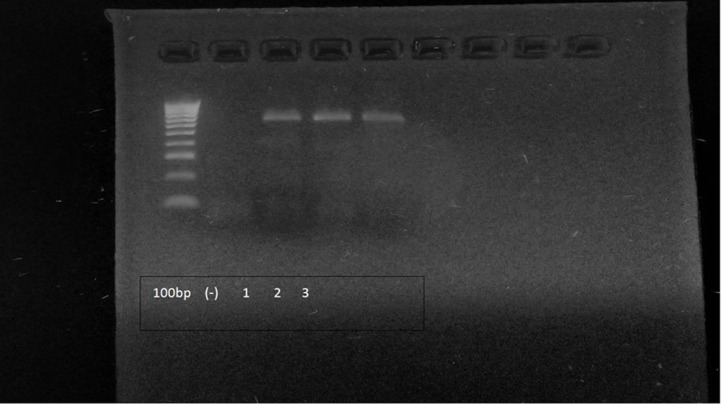
Visualization of PCR products for the detection of *P. destructans.* The image shows the negative control (-) and three positive reactions using genomic DNA extracted from the wings of bats with injuries. The fragment size corresponds to an expected product of 624 base pairs.

We captured and collected samples from 11 individuals of *M. velifer* (8), *D. azteca* (2), and *P. parnellii* (1), and 15 carcasses of *P. parnellii* (3), *D. rotundus* (2), *M. velifer* (1), *D. azteca* (4), *B. plicata* (3), and *A. geoffroyi* (2) inside the cave; the carcasses were found inside the cave, on the ground beneath areas where bats roost; 5 unidentified carcasses were disregarded. Molecular analysis of 10 samples (9 from captured individuals and one carcass) confirmed the presence of *P. destructans* with the amplification of a 624 bp fragment extracted from the DNA. The sequences of the amplified fragments were compared with the GeneBank database for *P. destructans* at the following percentages: (1) ID 99.17% with *P. destructans* MF681575.1; (3) ID 99.19% with *P. destructans* KP714633.1 and (1) ID 100% with *P. destructans* MK794130.1 (Fig 2). The molecular sequences databases obtained in the PCR analyses are included in the SI (S3 File).

Eight individuals of *M. velifer* were examined using the histopathological HE technique, of which seven individuals presented pathoanatomical changes. In five bats, severe lesions were observed, such as periadnexal pyogranulomatous dermatitis with the presence of intralesional conidia and ortho-keratotic hyperkeratosis with clustered hyphae (S4 File). In most specimens (N = 7), an inflammatory infiltration of lymphocytes and plasma cells was observed beneath the dermis and around the skin appendages (S4 File). At the same time, only one bat showed a loss of continuity (ulceration) in areas of the stratum corneum, replaced by cellular debris, degenerate neutrophils, lymphocytes, plasma cells and macrophages, with hair follicles distended by concentric layers of anucleate keratin.

Only species of vespertilionid and emballonurid bats tested positive for *P. destructans*: eight individual samples of individuals of *M. velifer* and one of *P. parnellii*. Of a total of 15 samples of carcasses, only one carcass of *B. plicata* tested positive for *P. destructans* (Table 1). Captured individuals testing positive for *P. destructans* showed either circular and separated or irregular and continuous lesions in the wings and body (S1 File). Individuals of *M. velifer* and *D. azteca* also showed fluorescence spots primarily in the wing membranes, patagium and rostrum. However, *D. azteca* did not test positive for *P. desctructans*. Other individuals showed lesions in the back and abdomen, while the carcasses were almost completely covered with the mycelium of the fungus (S1 File).

**Table 1:** Samples from live individuals (L) and carcasses (C) of different bat species tested positive (+) or negative (-) for *P. destructans* in 2018, 2022 and 2023. All bats were captured inside a cave located in Oaxaca, Mexico. The species and sex of individuals are listed. See Methods for details.

Sample	Species	Sex	*P. Destructans*	Year
L1	*Myotis velifer*	♂	**+**	2018
L2	*Myotis velifer*	♀	**+**	2018
L3	*Myotis velifer*	♀	**+**	2018
L4	*Myotis velifer*	♀	**+**	2018
L5	*Myotis velifer*	♀	**+**	2018
L6	*Myotis velifer*	♀	**+**	2018
L7	*Dermanura azteca*	♀	**–**	2018
L8	*Dermanura azteca*	♂	**–**	2018
L9	*Myotis velifer*	♂	**+**	2023
L10	*Myotis velifer*	♂	**+**	2023
L11	*Pteronotus parnellii*	♂	**+**	2023
C1	*Balantiopteryx plicata*	N/I	**+**	2022
C2	*Anoura geoffroyi*	N/I	**–**	2022
C3	*Anoura geoffroyi*	N/I	**–**	2022
C4	*Balantiopteryx plicata*	N/I	**–**	2022
C5	*Balantiopteryx plicata*	N/I	**–**	2022
C6	*Dermanura azteca*	N/I	**–**	2022
C7	*Dermanura azteca*	N/I	**–**	2022
C8	*Dermanura azteca*	N/I	**–**	2022
C9	*Dermanura azteca*	N/I	**–**	2022
C10	*Desmodus rotundus*	N/I	**–**	2022
C11	*Desmodus rotundus*	N/I	**–**	2022
C12	*Myotis velifer*	N/I	**–**	2023
C13	*Pteronotus parnellii*	N/I	**–**	2023
C14	*Pteronotus parnellii*	N/I	**–**	2023
15	*Pteronotus parnellii*	N/I	**–**	2023

**Fig 3:**
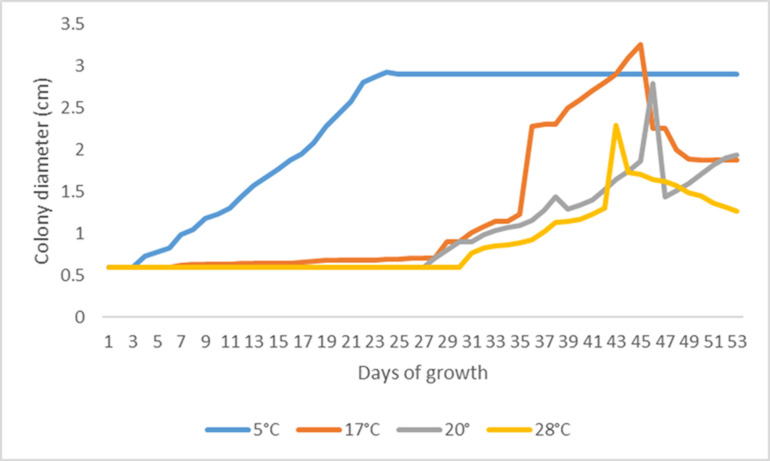
Thermotolerance growth curves of *P. destructans* at 5 °C (blue), 17 °C (red), 20 °C (gray) and 28 °C (yellow) after 53 days under laboratory conditions.

The thermotolerance test of *P. destructans* ATCC MYA 4855 strain confirmed that this fungal pathogen can grow at temperatures ranging from 5 °C–28 °C. The referenced strain grew fastest at 5 °C (from day 3–21), followed by 17 °C (from day 27–43), 20 °C (from day 27–45) and 28 °C (from day 28–43). All referenced strains grew to a colony diameter of 3 cm or higher, except for at 28 °C, which reached only 2 cm in diameter (Fig 3).

## Discussion

White-nose syndrome is an emerging disease caused by *P. destructans*, a fungal pathogen affecting the respiratory system with fatal consequences for several species of vespertilionid bats in North America [41,42]. Rapid colonization of large geographical areas of Asia, Europe and North America by *P. destructans* is facilitated by the long range of some bat species, particularly migratory species, as well as the movement of other host organisms, including humans [42–44]. So far, WNS appears to be present only in bats that are engaged in torpor and hibernating in underground sites and initially *P. destructans* appeared to grow optimally at only a small range of cold temperatures. However, thermotolerance tests performed with the *P. destructans* ATCC reference strain showed that the fungus can in fact grow at temperatures ranging from 5–28 °C (Fig 3), as observed in previous studies [23]; even the slower growth rate observed for the *P. destructans* ATCC reference strain at high temperatures could be harmful to hibernating bats that could allow for even small amounts of fungus to penetrate into their tissues. This wide temperature range could allow *P. destructans* to establish in a wide range of habitats inside underground sites in the Neotropics affecting bats [28,44]. Furthermore, our thermotolerance tests showed that culturing at 5 °C for approximately 23 days caused the *P. destructans* ATCC reference strain to stop growing, while at higher temperatures, its growth increased from 36 days reaching a maximum growth between 43 and 46 days (at 20 °C and 28 °C, respectively) (Fig 3). This could explain that bats occurring in Neotropical environmental conditions and underground sites showing warmer temperatures can still get infected by this fungal pathogen growing at higher temperatures.

Molecular analyses confirmed that there was a high similarity from field collected tissue samples with the ATCC MYA 4855 *P. destructans* reference strain. Further, fungal cultures obtained from carcasses were morphologically consistent with *P. destructans* strain although the morphology of the conidia was slightly different from the reference strain (S2 File), which could be caused by altered growth temperature [24]. When cultured at low (5 °C) temperatures, *P. destructans* conidia tend to be half-moon shaped, and when cultured at warmer temperatures (15 °C) they tend to present a globose morphology and become deformed at 18 °C [25]. The *P. destructans* that we collected from Oaxaca, Mexico, had globose morphology with hyphae forming chlamydospores (S2 File) resembling the morphology of other *Pseudogymnoascus* species at 28 °C.

Previous studies have shown that the cooler conditions of the Mexican highlands provide suitable environmental conditions for *P. destructans* [27,28]. In addition, ecological niche modeling has projected an overlap in distribution between species of vespertilionid bats and *P. destructans* across North America, Europe and Asia with 8 of the 26 species of bats sharing a similar projected environmental niche with the North American fungal strain of *P. destructans* in Mexico [27,28]. This modeling exercise suggested a high risk of *P. destructans* infection in bat populations and a high potential for fungal spread further south than the last official report in Texas [21,45]. Interestingly, a similarity in environmental niche between several species of bats and the North American fungal strain also overlapped in the highlands of Oaxaca, including the Kava Yuu Yavi cave [27,28]. Our study is the first to corroborate in the field the geographical model that places of *P. destructans* in Mexico and evidence infection in *M. velifer*, *B. plicata* and *P. parnellii* [21,22]. This result amplifies the importance of monitoring other areas of potential overlap between bats and *P. destructans* that have been identified in Mexico [27,28]. Specifically, we propose that adjacent areas to our study site in Oaxaca be monitored and included in a regional program aimed at detecting new areas of dispersal of this fungal pathogen.

Migratory bat species are likely serving to disperse *P*. *destructans* across North American countries. For example, *Lasiurus cinereus,* which has been identified as resistant to WNS infection in epizootic areas [44,46], migrates from Canada and the U.S. to Mexico flying >1000 km each year [47]; the abundant to Mexico *E. fuscus* bats have tested positive for WNS in the U.S., and can migrate long distances [47]; and the migratory *M. velifer,* which develops WNS [48]. Bat species have previously been shown to host and spread other diseases. For example, the fruit-eating bat *Artibeus jamaicensis* in southern Mexico has been observed to have leishmaniasis, lesions to the wings and uropatagium, caused by a *Leishmania mexicana* infection [49].

Humans have also been identified as carrying *P. destructans* spores from Europe to the U.S. [39]. The possibility of humans contaminating populations of bats with *P. destructans* across countries is particularly likely in tourist locations, like our study site [50]. For this reason, it is important to establish regulations for tourists visiting our study site because it has been evidenced to be infected with *P. destructans*, but also for other bat habitats that have not yet been investigated.

Previous studies modeled the possible entrance route of WNS into Mexico based on the environmental overlap between species of bats and *P. destructans* [27,28]. They reveal that the northeast is the more common dispersal route than the northwest. For example, in the Sierra Madre Oriental northeast highlands an overlap of 68% was identified, while in the Sierra Madre Occidental northwest highlands, an overlap of 34% was identified. Similarly, in bat species that are widely distributed, the northeast showed greater overlap (73%) than the northwest highlands (23%). Routes of entry extend into the Transvolcanic Belt in central Mexico and the southwest Sierra Madre del Sur highlands in southern Mexico, where our study site is located (Fig 1) [28]. Interestingly, it has been shown a widespread presence of hibernacula caves where many individuals of vespertilionid bats torpor in the Sierra Madre Oriental northeast highlands and the Transvolcanic Belt in central Mexico, coinciding with our modeled routes of entries of *P. destructans* in Mexico [51].

Further, our results corroborate that *P. destructans* can grow at higher temperatures, thriving inside (20.9 °C) and outside (19.5 °C) of the caves [19,23]. The temperature range in the thermotolerance *of P. destructans* means that it can disperse and establish across the Neotropics. Further research is needed to determine if this fungal strain of *P. destructans* is adapting to grow optimally at higher temperatures and establishing in new and warmer regions and if its growth is dependent on both temperature and humidity [52].

Our study found that only vespertilionid and emballonurid bats tested positive for *P. destructans*, while species of Phyllostomidae (*D. azteca*, and *A. geoffroyi*) and the vampire bat *D. rotundus* remained unaffected by *P. destructans* (Table 1); however, the fungus appeared evident on *D. azteca* under fluorescent light lamps. The reason why these bat species did not develop WNS from *P. destructans* but showed tolerance [53] requires further research. Individual *M. velifer*, *P. parnellii* and *B. plicata* bats from our study site that appeared to be uninfected may have the potential to infect other populations and species of hibernating vespertilionid and emballonurid bats. Further, the possibility of an infection of *P. destructans* in other species of bats (e.g., Phyllostomidae) cannot be excluded. *P. destructans* behaves as a primary pathogen and an opportunistic saprobe, meaning that it has the enzymatic apparatus and infection strategies to invade the intact surface of the skin and has an affinity for the hair follicle, which provides a suitable microenvironment for fungal germination [54]. In addition, other bat species are likely to visit underground sites and roosting sites where infected bats co-occur and interact. Although bats suffer from the effects of the fungus during hibernation, the fungus is likely to spread among species throughout their lifecycle, with many of them serving as dispersers of the fungus causing WNS [26].

This study evidencing the presence of *P. destructans* in bat species of Oaxaca, Mexico, evidences the need for environmental management to protect migratory and resident bat species against the pathogen *P. destructans,* which causes WNS. Precautions and actions are particularly important given that Oaxaca is considered a biodiversity hotspot of bats [29]. Bats play important roles in providing ecosystem services, such as pollinating and dispersing plant seeds and consuming large amounts of pest crop insects. Thus, we urge that a monitoring program in and adjacent to the study site be put in place to monitor the presence of WNS in bats. In addition, farmers and other stakeholders would benefit from understanding how a loss of bat populations will affect their crops and encourage a citizen digital reporting system where the presence of WNS infected bats or carcasses can be reported. In addition, because the expected thermotolerance of *P. destructans* [23] appears to be expanding, conservation managers should be on the lookout for *P. destructans* in previously unexpected areas. A multidisciplinary approach that includes studies focusing on the regional dispersal of this emerging disease, as well as molecular and morphological research, is the best defense against the loss of bat species due to WNS in the Neotropics.

## Supporting information

S1 File Presence of irregular ulcer lesions in wings, ears and alopecia in the shoulder, and lesions in ear of an individual *Myotis velifer* (A in both pictures). Note the color of the lesions in the wing and body, that corresponds to *P. destructans*. A carcass of an individual *Myotis velifer* showing acute mycosis. The mycelial masses shown are consistent with *P. destructans* growth (**B**).
(DOCX)

S2 File Images of *P. destructans* fungus strains obtained from our study site in Oaxaca, Mexico, showing globose morphology (A), and hyphae forming chlamydospores (B), resembling other *Pseudogymnoascus* species morphology at 28 °C (Photographs were taken with a Velab VE-BC3 Plus at 40 X). (DOCX)

S3 File Molecular sequences database obtained from the samples included in the PCR analyses. See Methods for details.
(DOCX)

S4 File Histopathological images of *P. destructans* fungus strain obtained from our study site in Oaxaca, Mexico, showing (A) intralesional conidia (red arrow) and abundant inflammatory infiltrate (black arrow), (B) presence of hyphae in clusters (red arrow), parallel layers of anucleate keratin (black arrow), and (C) presence of inflammatory infiltrate composed of lymphocytes and plasma cells interspersed with cellular debris. (DOCX)
