# Supplementary material for: Presence of white-nose syndrome in bats from Southern Mexico
Source: PLoS One. 2025 May 19;20(5):e0318461. doi: 10.1371/journal.pone.0318461 (PMC12088370; doi:10.1371/journal.pone.0318461)

**SI1**. Presence of irregular ulcer lesions in wings, ears and alopecia in the shoulder, and lesions in ear of an individual *Myotis velifer* (**A** in both pictures). Note the color of the lesions in the wing and body, that corresponds to *P. destructans*. A carcass of an individual *Myotis velifer* showing acute mycosis. The mycelial masses shown are consistent with *P. destructans* growth (**B**).

*.*
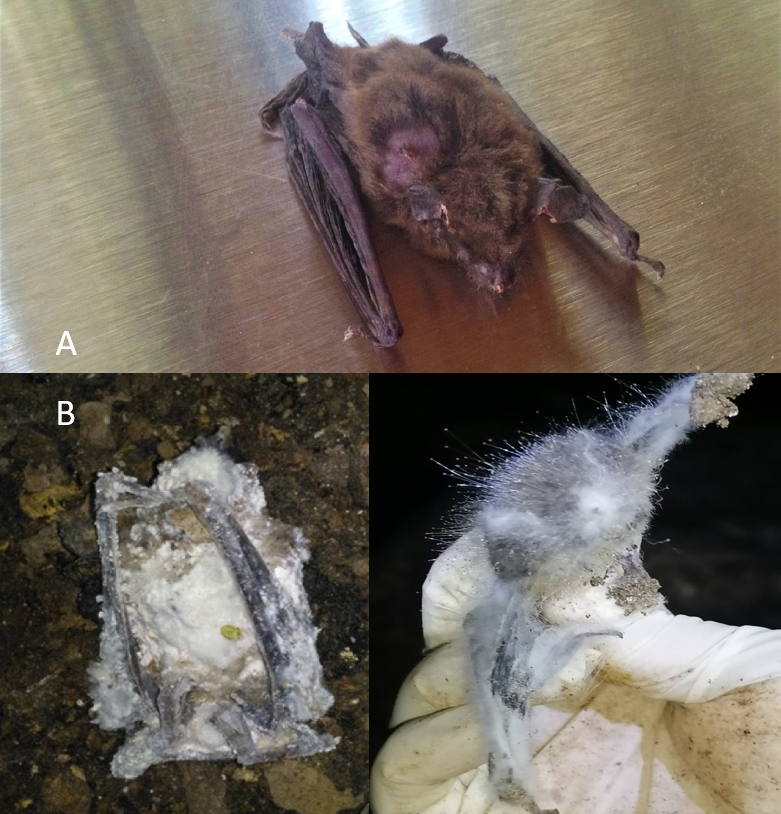

Supplement: S1 File — Note the color of the lesions in the wing and body, that corresponds to P. destructans. A carcass of an individual Myotis velifer showing acute mycosis. The mycelial masses shown are consistent with P. destructans growth (B). (DOCX) [file pone.0318461.s001.docx]
