# Supplementary material for: Presence of white-nose syndrome in bats from Southern Mexico
Source: PLoS One. 2025 May 19;20(5):e0318461. doi: 10.1371/journal.pone.0318461 (PMC12088370; doi:10.1371/journal.pone.0318461)

**SI2**. Images of *P. destructans* fungus strains obtained from our study site in Oaxaca, Mexico, showing globose morphology (**A**), and hyphae forming chlamydospores (**B**), resembling other *Pseudogymnoascus* species morphology at 28 °C (Photographs were taken with a Velab VE-BC3 Plus at 40 X).

**A**


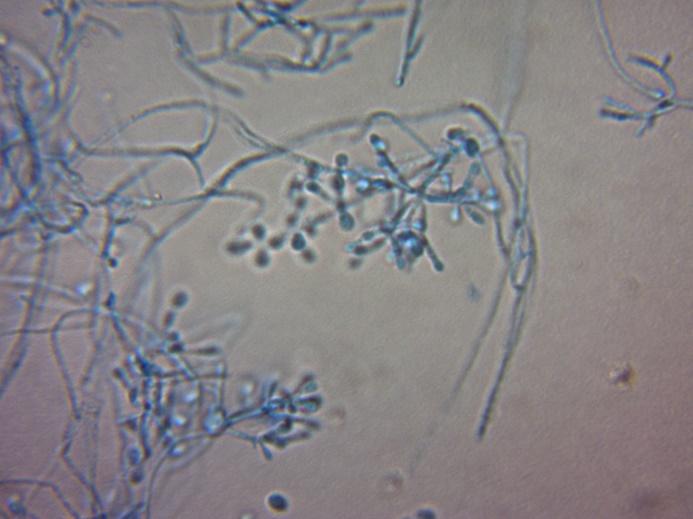


**B**


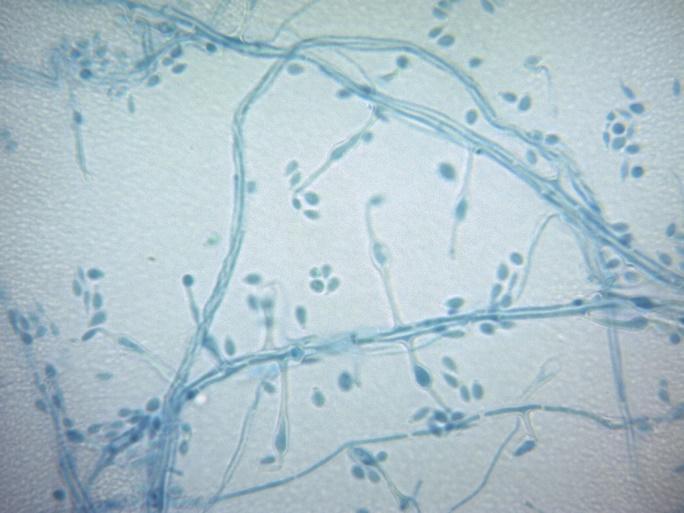

Supplement: S2 File — (DOCX) [file pone.0318461.s002.docx]
