# Supplementary material for: Presence of white-nose syndrome in bats from Southern Mexico
Source: PLoS One. 2025 May 19;20(5):e0318461. doi: 10.1371/journal.pone.0318461 (PMC12088370; doi:10.1371/journal.pone.0318461)
Supplement: S3 File — See Methods for details. (DOCX) [file pone.0318461.s003.docx]

**SI3** **(Database)**. Molecular sequences database obtained from the samples included in the PCR analyses. See Methods for details.

**>Sample C1. *Pseudogymnoascus destructans* isolate from *Balantiopteryx plicata* ribosomal RNA gene internal transcribed spacer (ITS) region (complete sequence)**

AAAGCCTACAACACCAACCTACCCGGGAAACCGAGGTAGGGGCCCGTGCTAACTACACGGGGTGGTAAAAGAATGTATGGATACTCCCTCCGGGGAACTATGGATAATCCGCAGCGAAGACCCTAAGTAGCGCTAGCTATACGGGTAACGTTCACAGACTAAGTGGTTGTGGGTGGAGCCTAGCTCTGCTTAAGATATAGTCGGGCCCTGCGTGAAAGCGCAGGGGTGAGTCGCTACGAACTCGAACCGTTCCGTAGGTGAACCTGCGGAAGGATCATTACAGTAGTCGCCCGGGTTGCCGCAAGGCCTCCCGGGTAACCTACCACCCTTTGTTTATTACACTTTGTTGCTTTGGCAGGCCTGCCCTCGGGCTGCTGGCTCCGGCCGGCGAGCGCTTGCCAGAGGACCTAAACTCTGTTTGTCTATACTGTCTGAGTACTATATAATAGTTAAAACTTTCAACAACGGATCTCTTGGTTCTGGCATCGATGAAGAACGCAGCGAAATGCGATAAGTAATGTGAATTGCAGAATTCAGTGAATCATCGAATCTTTGAACGCACATTGCGCCCCCTGGTATTCCGGGGGGCATGCCTGTCC

**>Sample C1. *Pseudogymnoascus destructans* isolate from *Balantiopteryx plicata* ribosomal RNA gene internal transcribed spacer (ITS) region (forward sequence)**

ACCCCAACCTACCCGGGAACCGAGGTAGGGGCCCGTGCTAACTACACGGGGTGGTAAAAGAATGTATGGATACTCCCTCCGGGGAACTATGGATAATCCGCAGCGAAGACCCTAAGTAGCGCTAGCTATACGGGTAACGTTCACAGACTAAGTGGTTGTGGGTGGAGCCTAGCTCTGCTTAAGATATAGTCGGGCCCTGCGTGAAAGCGCAGGGGTGAGTCGCTACGAACTCGAACCGTTCCGTAGGTGAACCTGCGGAAGGATCATTACAGTAGTCGCCCGGGTTGCCGCAAGGCCTCCCGGGTAACCTACCACCCTTTGTTTATTACACTTTGTTGCTTTGGCAGGCCTGCCCTCGGGCTGCTGGCTCCGGCCGGCGAGCGCTTGCCAGAGGACCTAAACTCTGTTTGTCTATACTGTCTGAGTACTATATAATAGTTAAAACTTTCAACAACGGATCTCTTGGTTCTGGCATCGATGAAGAACGCAGCGAAATGCGATAAGTAATGTGAATTGCATAATTCAATGAATCATCGAATCTTTGAACGCACATTGCGCCCCCTGGTATTCCGGGGGGCATGCCTGTCC

**>Sample C1. *Pseudogymnoascus destructans* isolate from *Balantiopteryx plicata* ribosomal RNA gene internal transcribed spacer (ITS) region (reverse sequence)**

AAAGCCTACAACACCAACCTACCCGGGAAACCGAGGTAGGGGCCCGTGCTAACTACACGGGGGTGGTAAAAGAATGTATGGATACTCCCTCCGGGGAACTATGGATAATCCGCAGCGAAGACCCTAAGTAGCGCTAGCTATACGGGTAACGTTCACAGACTAAGTGGTTGTGGGTGGAGCCTAGCTCTGCTTAAGATATAGTCGGGCCCTGCGTGAAAGCGCAGGGGTGAGTCGCTACGCACTCGAACCGTTCCGTAGGTGAACCTGCGGAAGGATCATTACAGTAGTCGCCCGGGTTGCCGCAAGGCCTCCCGGGTAACCTACCACCCTTTGTTTATTACACTTTGTTGCTTTGGCAGGCCTGCCCTCGGGCTGCTGGCTCCGGCCGGCGAGCGCTTGCCAGAGGACCTAAACTCTGTTTGTCTATACTGTCTGAGTACTATATAATAGTTAAAACTTTCAACAACGGATCTCTTGGTTCTGGCATCGATGAAGAACGCAGCGAAATGCGATAAGTAATGTGAATTGCAGAATTCAGTGAATCATCGAATCTTTGAACGCACATTGCGCCCCCTGGTATTCCGGGGGGCA

**>Sample L6. *Pseudogymnoascus destructans* isolate from *Myotis velifer* ribosomal RNA gene internal transcribed spacer (ITS) region (completed sequence)**

GGGGACGTCCTAAAGCCTACAACACCAACCTACCCGGGAAACCGAGGTAGGGGCCCGTGCTAACTACACGGGGTGGTAAAAGAATGTATGGATACTCCCTCCGGGGAACTATGGATAATCCGCAGCGAAGACCCTAAGTAGCGCTAGCTATACGGGTAACGTTCACAGACTAAGTGGTTGTGGGTGGAGCCTAGCTCTGCTTAAGATATAGTCGGGCCCTGCGTGAAAGCGCAGGGGTGAGTCGCTACGAACTCGAACCGTTCCGTAGGTGAACCTGCGGAAGGATCATTACAGTAGTCGCCCGGGTTGCCGCAAGGCCTCCCGGGTAACCTACCACCCTTTGTTTATTACACTTTGTTGCTTTGGCAGGCCTGCCCTCGGGCTGCTGGCTCCGGCCGGCGAGCGCTTGCCAGAGGACCTAAACTCTGTTTGTCTATACTGTCTGAGTACTATATAATAGTTAAAACTTTCAACAACGGATCTCTTGGTTCTGGCATCGATGAAGAACGCAGCGAAATGCGATAAGTAATGTGAATTGCAGAATTCAGTGAATCATCGAATCTTTGAACGCACATTGCGCCCCCTGGTATTCCGGGGGGCATGCCTGTCCGAGCGTCATTACAA

**>Sample L6. *Pseudogymnoascus destructans* isolate from *Myotis velifer* ribosomal RNA gene internal transcribed spacer (ITS) region (forward sequence)**

GGTAGGGGCCCGTGCTAACTACACGGGGTGGTAAAAGAATGTATGGATACTCCCTCCGGGGAACTATGGATAATCCGCAGCGAAGACCCTAAGTAGCGCTAGCTATACGGGTAACGTTCACAGACTAAGTGGTTGTGGGTGGAGCCTAGCTCTGCTTAAGATATAGTCGGGCCCTGCGTGAAAGCGCAGGGGTGAGTCGCTACGAACTCGAACCGTTCCGTAGGTGAACCTGCGGAAGGATCATTACAGTAGTCGCCCGGGTTGCCGCAAGGCCTCCCGGGTAACCTACCACCCTTTGTTTATTACACTTTGTTGCTTTGGCAGGCCTGCCCTCGGGCTGCTGGCTCCGGCCGGCGAGCGCTTGCCAGAGGACCTAAACTCTGTTTGTCTATACTGTCTGAGTACTATATAATAGTTAAAACTTTCAACAACGGATCTCTTGGTTCTGGCATCGATGAAGAACGCAGCGAAATGCGATAAGTAATGTGAATTGCAGAATTCAGTGAATCATCGAATCTTTGAACGCACATTGCGCCCCCTGGTATTCCGGGGGGCATGCCTGTCCGAG

**>Sample L6. *Pseudogymnoascus destructans* isolate from *Myotis velifer* ribosomal RNA gene internal transcribed spacer (ITS) region (reverse sequence)**

GGGGAYGTCCTAAAGCCTACAAACACCAACCTACCCGGGAAACCGAGGTAGGGGCCCGTGCTAACTACACGGGGTGGTAAAAGAATGTATGGATACTCCCTCCGGGGAACTATGGATAATCCGCAGCGAAGACCCTAAGTAGCGCTAGCTATACGGGTAACGTTCACAGACTAAGTGGTTGTGGGTGGAGCCTAGCTCTGCTTAAGATATAGTCGGGCCCTGCGTGAAAGCGCAGGGGTGAGTCGCTACGAACTCGAACCGTTCCGTAGGTGAACCTGCGGAAGGATCATTACAGTAGTCGCCCGGGTTGCCGCAAGGCCTCCCGGGTAACCTACCACCCTTTGTTTATTACACTTTGTTGCTTTGGCAGGCCTGCCCTCGGGCTGCTGGCTCCGGCCGGCGAGCGCTTGCCAGAGGACCTAAACTCTGTTTGTCTATACTGTCTGAGTACTATATAATAGTTAAAACTTTCAACAACGGATCTCTTGGTTCTGGCATCGATGAAGAACGCAGCGAAATGCGATAAGTAATGTGAATTGCAGAATTCAGTGAATCATTCGAATCTTTGAACGCACATTGCGCCCCC

**>Sample L9. *Pseudogymnoascus destructans* isolate from *Myotis velifer* ribosomal RNA gene internal transcribed spacer (ITS) region (completed sequence)**

CTAAAGCCTACAACACCAACCTACCCGGGAAACCGAGGTAGGGGCCCGTGCTAACTACACGGGGTGGTAAAAGAATGTATGGATACTCCCTCCGGGGAACTATGGATAATCCGCAGCGAAGACCCTAAGTAGCGCTAGCTATACGGGTAACGTTCACAGACTAAGTGGTTGTGGGTGGAGCCTAGCTCTGCTTAAGATATAGTCGGGCCCTGCGTGAAAGCGCAGGGGTGAGTCGCTACGAACTCGAACCGTTCCGTAGGTGAACCTGCGGAAGGATCATTACAGTAGTCGCCCGGGTTGCCGCAAGGCCTCCCGGGTAACCTACCACCCTTTGTTTATTACACTTTGTTGCTTTGGCAGGCCTGCCCTCGGGCTGCTGGCTCCGGCCGGCGAGCGCTTGCCAGAGGACCTAAACTCTGTTTGTCTATACTGTCTGAGTACTATATAATAGTTAAAACTTTCAACAACGGATCTCTTGGTTCTGGCATCGATGAAGAACGCAGCGAAATGCGATAAGTAATGTGAATTGCAGAATTCAGTGAATCATCGAATCTTTGAACGCACATTGCGCCCCCTGGTATTCCGGGGGGCATGCCTGTCCGAGCGTCATTACAA

**>Sample L9. *Pseudogymnoascus destructans* isolate from *Myotis velifer* ribosomal RNA gene internal transcribed spacer (ITS) region (forward sequence)**

CCAACCTACCCGGGAAACCGAGGTAGGGGCCCGTGCTAACTACACGGGGTGGTAAAAGAATGTATGGATACTCCCTCCGGGGAACTATGGATAATCCGCAGCGAAGACCCTAAGTAGCGCTAGCTATACGGGTAACGTTCACAGACTAAGTGGTTGTGGGTGGAGCCTAGCTCTGCTTAAGATATAGTCGGGCCCTGCGTGAAAGCGCAGGGGTGAGTCGCTACGAACTCGAACCGTTCCGTAGGTGAACCTGCGGAAGGATCATTACAGTAGTCGCCCGGGTTGCCGCAAGGCCTCCCGGGTAACCTACCACCCTTTGTTTATTACACTTTGTTGCTTTGGCAGGCCTGCCCTCGGGCTGCTGGCTCCGGCCGGCGAGCGCTTGCCAGAGGACCTAAACTCTGTTTGTCTATACTGTCTGAGTACTATATAATAGTTAAAACTTTCAACAACGGATCTCTTGGTTCTGGCATCGATGAAGAACGCAGCGAAATGCGATAAGTAATGTGAATTGCAGAATTCAGTGAATCATCGAATCTTTGAACGCACATTGCGCCCCCTGGTATTCCGGGGGGCATGCCTGTCCGAGCGTCATTACAA

**>Sample L9. *Pseudogymnoascus destructans* isolate from *Myotis velifer* ribosomal RNA gene internal transcribed spacer (ITS) region (reverse sequence)**

CTAAAGCCTACAACACCAACCTACCCGGGAAACCGAGGTAGGGGCCCGTGCTAACTACACGGGGTGGTAAAAGAATGTATGGATACTCCCTCCGGGGAACTATGGATAATCCGCAGCGAAGACCCTAAGTAGCGCTAGCTATACGGGTAACGTTCACAGACTAAGTGGTTGTGGGTGGAGCCTAGCTCTGCTTAAGATATAGTCGGGCCCTGCGTGAAAGCGCAGGGGTGAGTCGCTACGAACTCGAACCGTTCCGTAGGTGAACCTGCGGAAGGATCATTACAGTAGTCGCCCGGGTTGCCGCAAGGCCTCCCGGGTAACCTACCACCCTTTGTTTATTACACTTTGTTGCTTTGGCAGGCCTGCCCTCGGGCTGCTGGCTCCGGCCGGCGAGCGCTTGCCAGAGGACCTAAACTCTGTTTGTCTATACTGTCTGAGTACTATATAATAGTTAAAACTTTCAACAACGGATCTCTTGGTTCTGGCATCGATGAAGAACGCAGCGAAATGCGATAAGTAATGTGAATTGCAGAATTCAGTGAATCATCGAATCTTTGAACGCACATTGCGCCCCCTGGTATTCCGGGGGGC

**>Sample L10. *Pseudogymnoascus destructans* isolate from *Myotis velifer* ribosomal RNA gene internal transcribed spacer (ITS) region (complete sequence)**

CTAAAGCCTACAACACCAACCTACCCGGGAAACCGAGGTAGGGGCCCGTGCTAACTACACGGGGTGGTAAAAGAATGTATGGATACTCCCTCCGGGGAACTATGGATAATCCGCAGCGAAGACCCTAAGTAGCGCTAGCTATACGGGTAACGTTCACAGACTAAGTGGTTGTGGGTGGAGCCTAGCTCTGCTTAAGATATAGTCGGGCCCTGCGTGAAAGCGCAGGGGTGAGTCGCTACGAACTCGAACCGTTCCGTAGGTGAACCTGCGGAAGGATCATTACAGTAGTCGCCCGGGTTGCCGCAAGGCCTCCCGGGTAACCTACCACCCTTTGTTTATTACACTTTGTTGCTTTGGCAGGCCTGCCCTCGGGCTGCTGGCTCCGGCCGGCGAGCGCTTGCCAGAGGACCTAAACTCTGTTTGTCTATACTGTCTGAGTACTATATAATAGTTAAAACTTTCAACAACGGATCTCTTGGTTCTGGCATCGATGAAGAACGCAGCGAAATGCGATAAGTAATGTGAATTGCAGAATTCAGTGAATCATCGAATCTTTGAACGCACATTGCGCCCCCTGGTATTCCGGGGGGCATGCCTGTCCGAGCGTCATTACAA

**>Sample L10. *Pseudogymnoascus destructans* isolate from *Myotis velifer* ribosomal RNA gene internal transcribed spacer (ITS) region (forward sequence)**

CCGGGAAACCGAGGTAGGGGCCCGTGCTAACTACACGGGGTGGTAAAAGAATGTATGGATACTCCCTCCGGGGAACTATGGATAATCCGCAGCGAAGACCCTAAGTAGCGCTAGCTATACGGGTAACGTTCACAGACTAAGTGGTTGTGGGTGGAGCCTAGCTCTGCTTAAGATATAGTCGGGCCCTGCGTGAAAGCGCAGGGGTGAGTCGCTACGAACTCGAACCGTTCCGTAGGTGAACCTGCGGAAGGATCATTACAGTAGTCGCCCGGGTTGCCGCAAGGCCTCCCGGGTAACCTACCACCCTTTGTTTATTACACTTTGTTGCTTTGGCAGGCCTGCCCTCGGGCTGCTGGCTCCGGCCGGCGAGCGCTTGCCAGAGGACCTAAACTCTGTTTGTCTATACTGTCTGAGTACTATATAATAGTTAAAACTTTCAACAACGGATCTCTTGGTTCTGGCATCGATGAAGAACGCAGCGAAATGCGATAAGTAATGTGAATTGCAGAATTCAGTGAATCATCGAATCTTTGAACGCACATTGCGCCCCCTGGTATTCCGGGGGGCATGCCTGTCCGAGCGTCATTACAA

**>Sample L10. *Pseudogymnoascus destructans* isolate from *Myotis velifer* ribosomal RNA gene internal transcribed spacer (ITS) region (reverse sequence)**

CTAAAGCCTACAACACCAACCTACCCGGGAAACCGAGGTAGGGGCCCGTGCTAACTACACGGGGTGGTAAAAGAATGTATGGATACTCCCTCCGGGGAACTATGGATAATCCGCAGCGAAGACCCTAAGTAGCGCTAGCTATACGGGTAACGTTCACAGACTAAGTGGTTGTGGGTGGAGCCTAGCTCTGCTTAAGATATAGTCGGGCCCTGCGTGAAAGCGCAGGGGTGAGTCGCTACGAACTCGAACCGTTCCGTAGGTGAACCTGCGGAAGGATCATTACAGTAGTCGCCCGGGTTGCCGCAAGGCCTCCCGGGTAACCTACCACCCTTTGTTTATTACACTTTGTTGCTTTGGCAGGCCTGCCCTCGGGCTGCTGGCTCCGGCCGGCGAGCGCTTGCCAGAGGACCTAAACTCTGTTTGTCTATACTGTCTGAGTACTATATAATAGTTAAAACTTTCAACAACGGATCTCTTGGTTCTGGCATCGATGAAGAACGCAGCGAAATGCGATAAGTAATGTGAATTGCAGAATTCAGTGAATCATCGAATCTTTGAACGCACATTGCGCCCCCTGGTATTCCGGGGGGC

**>Sample L11. *Pseudogymnoascus destructans* isolate from *Pteronotus parnellii* ribosomal RNA gene internal transcribed spacer (ITS) region (complete sequence)**

CTAAAGCCTACAACACCAACCCGCCCGGGAAACCGAGGCGGGGGCCCGTGCTAACTCCACGGGGTGGTAAAAGAGTGTATGGATACTCCCTCTGGGGAACTATGGATAATCCGCAGCGAAGACCCTAAGTAGCGCTAGCTATACGGGTAACGTTCACAGACTAAGTGGTTGTGGGTGGAGCCTAGCTCTGCTTAAGATATAGTCGGGCCCTACGTGAAAGCGCAGGGGTGAGTCGCTACGAACTCGAAACCGTTCCGTAGGTGAACCTGCGGAAGGATCATTACAGTAGTCGCCCGGGTTGCCGCAAGGCCTCCCGGGTAACCTACCACCCTTTGTTTATTACACTTTGTTGCTTTGGCAGGCCTGCCCTCGGGCTGCTGGCTCCGGCCGGCGAGCGCTTGCCAGAGGACTAAACTCTGTTTGTCTATACTGTCTGAGTACTATATAATAGTTAAAACTTTCAACAACGGATCTCTTGGTTCTGGCATCGATGAAGAACGCAGCGAAATGCGATAAGTAATGTGAATTGCAGAATTCAGTGAATCATCGAATCTTTGAACGCACATTGCGCCCCCTGGTATTCCGGGGGGCATGCCTGTCCGAGCGTCATTACAA

**>Sample L11. *Pseudogymnoascus destructans* isolate from *Pteronotus parnellii* ribosomal RNA gene internal transcribed spacer (ITS) region (forward sequence)**

AGGCGGGGGCCCGTGCTAACTCCACGGGGTGGTAAAAGAGTGTATGGATACTCCCTCTGGGGAACTATGGATAATCCGCAGCGAAGACCCTAAGTAGCGCTAGCTATACGGGTAACGTTCACAGACTAAGTGGTTGTGGGTGGAGCCTAGCTCTGCTTAAGATATAGTCGGGCCCTACGTGAAAGCGCAGGGGTGAGTCGCTACGAACTCGAAACCGTTCCGTAGGTGAACCTGCGGAAGGATCATTACAGTAGTCGCCCGGGTTGCCGCAAGGCCTCCCGGGTAACCTACCACCCTTTGTTTATTACACTTTGTTGCTTTGGCAGGCCTGCCCTCGGGCTGCTGGCTCCGGCCGGCGAGCGCTTGCCAGAGGACTAAACTCTGTTTGTCTATACTGTCTGAGTACTATATAATAGTTAAAACTTTCAACAACGGATCTCTTGGTTCTGGCATCGATGAAGAACGCAGCGAAATGCGATAAGTAATGTGAATTGCAGAATTCAGTGAATCATCGAATCTTTGAACGCACATTGCGCCCCCTGGTATTCCGGGGGGCATGCCTGTCCGAGCGTCATTACAA

**>Sample L11. *Pseudogymnoascus destructans* isolate from *Pteronotus parnellii* ribosomal RNA gene internal transcribed spacer (ITS) region (reverse sequence)**

CTAAAGCCTACAACACCAACCCGCCCGGGAAACCGAGGCGGGGGCCCGTGCTAACTCCACGGGGTGGTAAAAGAGTGTATGGATACTCCCTCTGGGGAACTATGGATAATCCGCAGCGAAGACCCTAAGTAGCGCTAGCTATACGGGTAACGTTCACAGACTAAGTGGTTGTGGGTGGAGCCTAGCTCTGCTTAAGATATAGTCGGGCCCTACGTGAAAGCGCAGGGGTGAGTCGCTACGAACTCGAAACCGTTCCGTAGGTGAACCTGCGGAAGGATCATTACAGTAGTCGCCCGGGTTGCCGCAAGGCCTCCCGGGTAACCTACCACCCTTTGTTTATTACACTTTGTTGCTTTGGCAGGCCTGCCCTCGGGCTGCTGGCTCCGGCCGGCGAGCGCTTGCCAGAGGACTAAACTCTGTTTGTCTATACTGTCTGAGTACTATATAATAGTTAAAACTTTCAACAACGGATCTCTTGGTTCTGGCATCGATGAAGAACGCAGCGAAATGCGATAAGTAATGTGAATTGCAGAATTCAGTGAATCATCGAATCTTTGAACGCACATTGCGCCCCCTGGTATTCCGGGGGGC
