# Supplementary material for: Presence of white-nose syndrome in bats from Southern Mexico
Source: PLoS One. 2025 May 19;20(5):e0318461. doi: 10.1371/journal.pone.0318461 (PMC12088370; doi:10.1371/journal.pone.0318461)

**SI4**. Histopathological images of *P. destructans* fungus strain obtained from our study site in Oaxaca, Mexico, showing (**A**) intralesional conidia (red arrow) and abundant inflammatory infiltrate (black arrow), (**B**) presence of hyphae in clusters (red arrow), parallel layers of anucleate keratin (black arrow), and (**C**) presence of inflammatory infiltrate composed of lymphocytes and plasma cells interspersed with cellular debris.


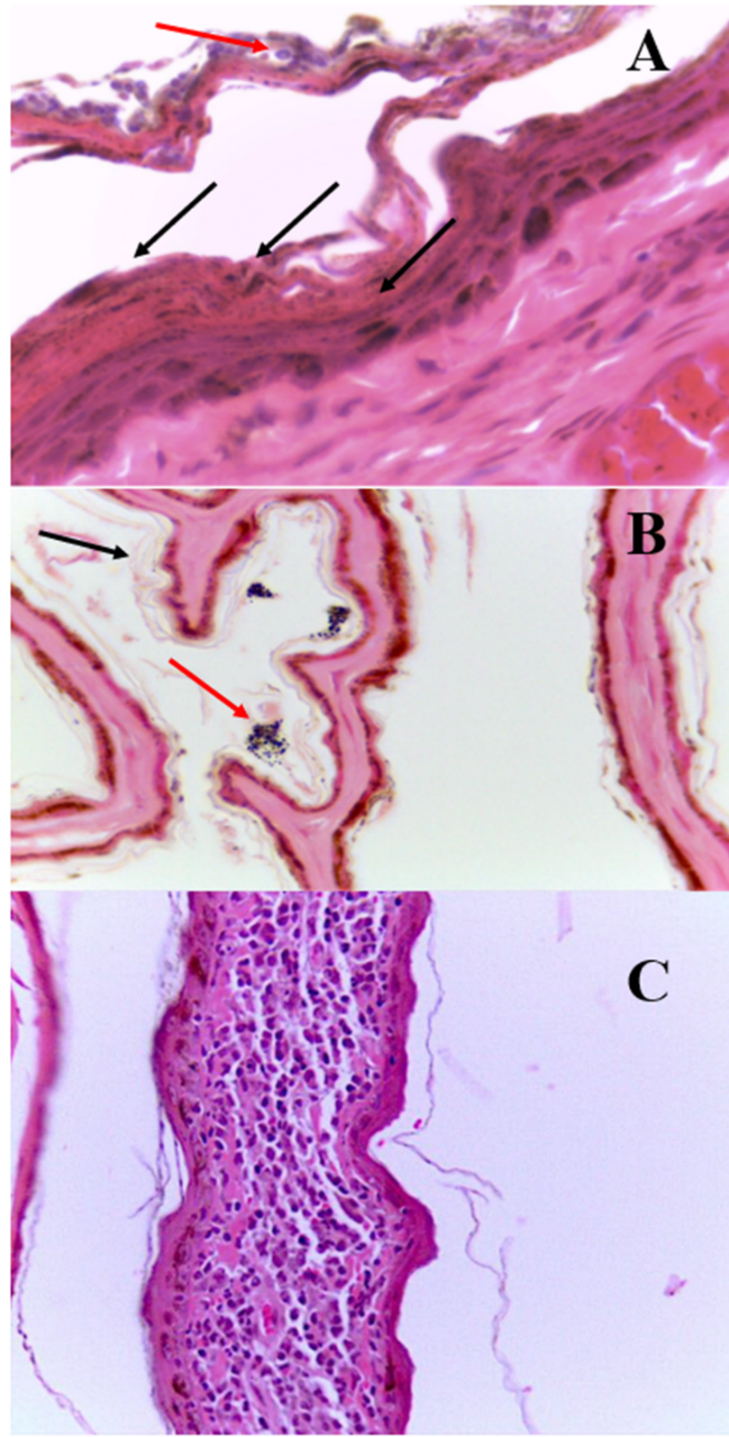

Supplement: S4 File — (DOCX) [file pone.0318461.s004.docx]
